# Supplementary figures and images for: Ketamine and sleep modulate neural complexity dynamics in cats
Source: Eur J Neurosci. 2022 Mar 19;55(6):1584–600. doi: 10.1111/ejn.15646 (PMC9310726; doi:10.1111/ejn.15646)

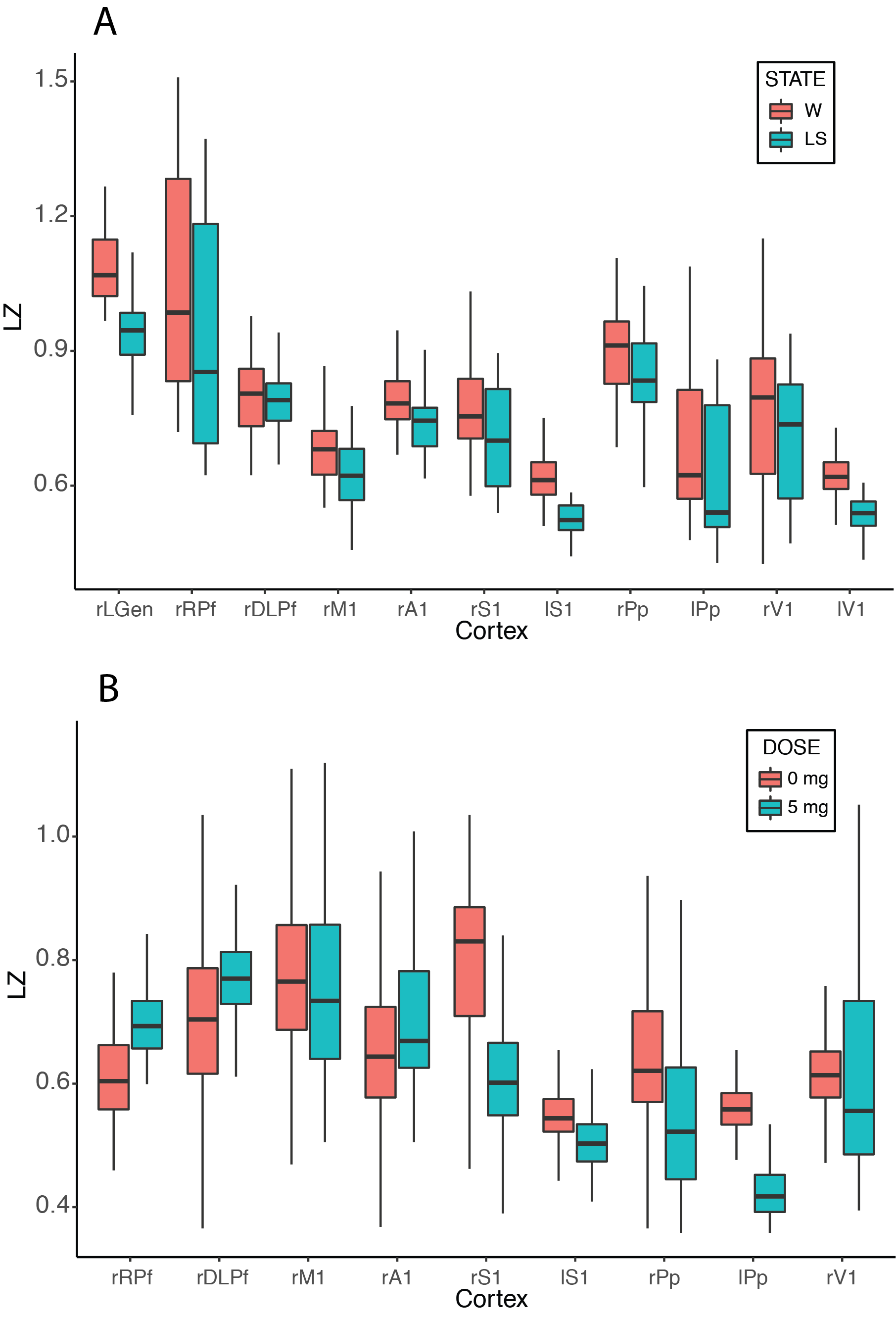

Supplement: Supplementary file 1 — FIGURE S1. Comparison between LZ in wakefulness and LS (a), and wakefulness without ketamine vs. 5 mg of Ketamine (b) for the different cortices. RPf, rostral prefrontal cortex; DLPf, dorsolateral prefrontal cortex; M, primary motor cortex; S, primary somatosensory cortex; A, primary auditory cortex, Pp, posterior parietal cortex; V, visual cortex. “l” indicates left side, “r” right side and “1” indicates “primary cortex”. [file EJN-55-1584-s001.png]
